# Alternatives to Conventional Evaluation of Rideability in Horse Performance Tests: Suitability of Rein Tension and Behavioural Parameters

**DOI:** 10.1371/journal.pone.0087285

**Published:** 2014-01-29

**Authors:** Uta König von Borstel, Chantal Glißman

**Affiliations:** Department of Animal Science, University of Göttingen, Göttingen, Germany; University of Rennes 1, France

## Abstract

Rideability, i.e. the ease and comfort with which a horse can be ridden, is considered to be one of the most important traits in riding horses. However, at present rideability is evaluated rather subjectively in breeding horse performance tests. The aim of the present study was to evaluate the role horse behaviour as well as degree and quality of rein tension might play in judges’ evaluation of horses’ rideability. Mares (n = 33) and stallions (n = 13) from two different mare- and one stallion-testing station were observed twice during their performance test dressage training. During these rides, rein tension was measured continuously, and frequency of behaviour patterns such as head-tossing, tail swishing, and snorting was recorded. Rein tension parameters showed reasonable repeatabilities within horse-rider pairs (e.g. mean rein tension: r^2^ = 0.61±0.11; variance of rein tension: r^2^ = 0.52±0.14). Regression analysis revealed that a larger proportion of variance in rideability scores could be explained by maximum (17%), mean (16%) and variance (15%) of rein tension compared to horses’ or riders’ behavioural parameters (tail-swishing: 5% and rider’s use of hands: 5%, respectively). According to mixed model analysis, rideability scores dropped (all P<0.05) with increasing mean, maximum and variability in rein tension (e.g. −0.37±0.14 scores per additional 10 Newton in mean tension). However, mean rein tension differed between testing stations (P<0.0001) ranging between 9.1±1.6 N in one station and 21.7±1.3 N in another station. These results indicate that quantity and consistency of rein tension is either directly or indirectly an important factor for judges to derive rideability scores. Given the importance of rein tension parameters to both rider comfort and horse welfare, potentially, measurements of rein tension along with behaviour observations assessing the quality of rein contact (e.g. distinguishing a light contact from attempts to evade contact) might be used to make the assessment of rideability more impartial.

## Introduction

Rideability is a trait evaluated in a large proportion of horse breeding programmes (e.g. [1]). Rideability describes the degree of comfort a rider feels when riding a horse and the ease with which a horse can be ridden [2]–[4]. Although the level and quality of prior training can considerably influence rideability [4], during performance tests judges aim at assessing the horses’ innate aptitude rather than the level of rideability achieved by training. Besides the rider’s legs, seat and in some cases the voice, the reins are one of the main means of communication between rider and horse, and it is thus expected that the horse’s reaction to rider’s cues via these different channels largely determines its rideablity. The trait rideability is rated to be one of the most important traits by both riders [5] and breeders [6] of various riding horse breeds. Breeding associations take great efforts to evaluate the trait rideability. Exclusively for the trait rideability, external, independent test-riders are hired for each performance test to ride and evaluate the horses in addition to the evaluations taken in the training and test under the regular riders. However, based on the above definition, it becomes obvious that rideability is a very complex trait. Furthermore, a rider’s “feeling” is by definition a subjective experience, making the evaluation of this trait subjective. Although traditionally considered to be a performance rather than a personality trait [3], it is suggested that a horse’s rideability is also largely influenced by its sensitivity to the rider’s aids, its inclination to behave in certain ways and thus by its personality [4]. Learning ability, i.e. a component of personality, is also suggested to contribute to rideability [7]. Indeed many officially appointed judges and breeding authorities consider rideability to be a personality trait, too [4]. Therefore, rideability likely is a compound trait that comprises both conformation and personality aspects. For example, it can be assumed that a well-balanced horse, with long pasterns, a long neck and a slender throatlatch will likely be able to respond quickly and correctly to its rider’s requests, make the rider feel comfortable due to smooth gaits, and be physically less able to resist rein pressure. In the same way, a horse that is based on its genetic predisposition relaxed, with a medium sensitivity to tactile stimuli and that is quick to learn and to respond to riders’ aids will be supple and likewise be more comfortable to ride compared to one that is slow in learning and responding, tense, or that frequently spooks due to heightened fearfulness. As frequently pointed out, there is considerable room for improvement with personality trait evaluation in sport horse breeding programmes [8]–[12]. Rideability and personality traits are evaluated in breeding programmes based on subjective assessment methods [1], [12], leading to inflated and biased scores with limited variation between individuals [12], [13]. Based on these scores, a genetic selection appears to be unavailing. Perhaps due to its mixed status as a trait partially influenced by personality aspects and partially influenced by conformation aspects, the statistical distribution of rideability scores exhibits slightly more desirable properties compared to the conventional personality traits [12]. Nevertheless, more objective assessment methods could greatly improve the evaluation of rideability, ultimately enhancing genetic progress in this trait. For personality traits such as temperament or specifically fear reactivity, considerable effort has been put into the development of more objective assessment methods (see [14] for a review) such as direct behavioural observation during novel object tests [8], [10], [12], [15], [16], different riding situations [9], [17], handling situations [18] or veterinary inspections [19], [20]. However, with few recent exceptions [21], [22] comparably little attention has been paid to the trait rideability. Therefore, the objectives of the present work were to assess the relationship between conventional rideability scores and objective parameters, including the measurement of behaviour and rein tension, thereby providing insight into the mechanisms judges use to derive their evaluations of rideability. Furthermore, based on these relationships as well as repeatabilities, the suitability of these measurements for future, more objective rideability evaluation methods will be evaluated.

## Materials and Methods

This type of non-invasive, behavioural research is approved under the German animal protection act and does not require a study-specific permission. Owners (privately owned horses) or chief trainers of the testing stations (horses owned by the state stud) volunteered their horses to participate in the study. Except for equipping the horses with the rein tension device in addition to their normal tack all testing corresponded to the routine training procedures.

### Animals and Testing Conditions

A total of 46 German Riding Horses were observed for the present study. The majority of horses (n = 43) was the offspring of stallions licensed by one German breeding association, while three horses were the offspring of stallions licensed by another German breeding associations. All horses were participants of on-station mare (n = 2 stations; n = 33 mares) or stallion (n = 1 station; n = 13 stallions) performance tests, and they were either three (n = 35) or four (n = 11) years of age (table 1). They were housed for the duration of the performance test (mares: 4 weeks; stallions: 10 weeks) at the testing station in ca. 3×4 m individual box stalls with automatic drinkers and trained and/or turned out daily by the staff of the testing stations. Performance test guidelines suggest that horses should be well accustomed to carrying a saddle and a rider when entering the performance test. However, the level of training prior to the performance test remains at the discretion of the owners and was not known for the individual horses. During these on-station performance tests, horses are trained to be ridden in dressage and show-jumping and are evaluated for the basic gaits, jumping ability, rideability and the personality traits labelled “character”, “temperament” and “willingness to work” as well as “constitution” (stallion performance test only). Each trait is comprised of a variety of factors as described in more detail in the official evaluation guidelines [3] as well as previous studies [4], [23]. For example, rideability is supposed to be comprised of the rhythm and elasticity of the horse’s movement, its suppleness, posture, balance, reaction to the rider’s aids, the degree of chewing the bit, rein contact and the degree of comfort for the rider [3]. Each trait is graded on a scale from 1 (very poor) to 10 (excellent). Evaluation of these traits ensues in three steps: In the first step, the stations’ head coach grades the horses considering their performance during the 20–70 day training phase. In the second step, rideability, the gaits and jumping ability, but not the personality traits, are evaluated with the same scoring system in a 1-day final test, by a pair of external, certified judges appointed by the national equestrian federation. During the dressage test and the free-jumping parts of the final test horses are present by their regular rider they are familiar with from the training phase of the performance test. Also during this final test, in a third step an additional judge, i.e. a test rider likewise appointed by the national equestrian federation rides each horse for a brief period of 2–5 minutes in order to assign a third score for rideability only. Thus, rideability is judged twice by judges from the ground observing the horses’ performance under their familiar rider, and once by a judge mounting the horse, directly evaluating how easy and comfortable the horse is to ride. Horses’ final scores for rideability are the arithmetic mean of the scores from the three steps. Horses’ final scores for the performance traits are calculated by assigning double weight to scores from the final test and subsequently taking the mean of the training and final test scores. Horses were ridden during training and the final test by 15 different riders with a maximum of 8 horses per rider (n = 1 rider) and a minimum of 1 horse per rider (n = 4 riders). The judges differed for the three testing stations.

**Table 1:** Overview of total number (n) of horses and horses‘ gender, mean age, number of horses per age class (3 years [yrs] or 4 years old) and mean number of horses per rider by location.

Location	n	gender	mean age ± SD	n 3 yrs old	n 4 yrs old	Mean number of horses per rider
A	13	stallions	3.1±0.3	12	1	2.2
B	9	mares	3.1±0.3	8	1	2.1
C	24	mares	3.4±0.5	15	9	6.0

In all cases, dressage training was conducted in groups of three to five horses ridden simultaneously in indoor riding arenas measuring at least 20 × 40 m. Training was conducted for a mean (±SD) duration of 17.3±3.3 min. per day and included basic exercises such as transitions between gaits, and riding of simple dressage figures, such as circles or patterns leading to a change in hand.

### Data Collection

Each horse was observed twice during two dressage training sessions of the performance test. In accordance with an earlier study [9], the frequency of different behaviour patterns in both horse and rider were recorded per ride and converted on a per-hour-basis (table 2). In order to minimize subjectivity by introducing additional interpretations, behaviour patterns were recorded irrespective of their context. Teeth-grinding was observed, but not included in the further analysis due to occurrence in just two horses. Crabbing was likewise included in the ethogram, but no instances were observed. In contrast to the earlier study [9], observations were taken live, and only one horse at a time was observed. In addition, the observed horse was equipped during both rides with a rein tension meter (Signal Scribe, Crafted Technology, Australia), and rein tension was recorded to the inbuilt data logger. Before and after the full set of measurements per location, sensors of the rein tension device were calibrated using weights of 0.5, 1.0 and 2.0 kg, and no creep was detected. Official scores for all traits evaluated in the performance test were obtained from the testing stations after conclusion of the test.

**Table 2:** List and description of observed behaviour patterns recorded in frequencies of occurrence in the horse and rider (adapted from [9]).

Parameter	Description
**Horse related**	
Change in pace	The rhythm of a gait is interrupted because the horse attempts to change into a faster or slower gait
Snorting	The horse exhales air forcefully, producing a snoring sound
Tail-swishing	The tail moves horizontally (other, e.g. vertical movement disregarded)
Head-tossing	The horse throws its head forcefully upwards and/or sideways, pushing against the reins
Crabbing	The horse moves sideways, attempting to evade taking up additional load with the hind legs
Attempted buck	The horse arches the back and jumps with both hind legs upwards. While lowering the head. The motion is not performed at the maximum possible power (as in a complete buck) such that horses’ hind legs do not leave the ground simultaneously by more than 0.5 m
Stumbling	The rhythm of a gait is lost, often (but not limited to) because the horse hits its own leg with another leg
Shying	The horses shows a startle reaction with a subsequent attempt to flee
Teeth-grinding	The horse produces a sound by moving its teeth of upper and lower jaw with pressure against each other
**Rider related**	
Hand aids	The rider visibly moves the hand in relation to the body
Leg aids	The rider touches the horse with the whip
Use of whip	The rider visibly applies pressure with legs on the horse
Use of voice	The rider talks to the horse either in a calming voice or in an attempt to urge/reprimand the horse

### Statistical Analysis

Rein tension data was processed with the manufacturer-specific software and later analyzed along with the behavioural data and scores from performance tests using SAS (version 9.2). Mean, maximum and variance of rein tension was calculated for each ride separately, but combined for the right and left rein, and in addition, the absolute and relative difference between left and right rein mean tension was calculated as a measure of asymmetry of the horse-rider pair. All data were tested for distribution using the procedure UNIVARIATE. Traits that did not resemble a normal distribution (Kolmogorov-Smirnov: P<0.01) were analysed assuming either a Poisson distribution or, in the case of rare occurrences (i.e. occurrences in less than 15 rides and no more than 3 occurrences per ride) data were converted into binary data (e.g. bucking/no bucking observed per ride; see table 3).

**Table 3:** Frequencies per hour of riding of observed behaviour patterns as well as the type of data distribution assumed for analysis and the respective repeatabilities (±SE) of behaviour and rein tension parameters considering either the horse-rider dyad or rider only as random factor.

Parameter	Frequency per h ± SE	Data distribution	Repeatability (±SE) at the	
Horse related			Horse-rider level	Rider level
Change in pace	5.91±3.39	Poisson	0.14±0.07	0.07±0.05
Tail-swishing	4.91±0.91	Poisson	0.10±0.07	0.01±0.07
Head-tossing	8.82±1.83	Poisson	0.14±0.08	0.01±0.04
Attempted buck	0.75±0.30	binary	0.99±0.004	not converged
Snorting	1.50±0.37	binary	0.90±0.04	0.89±0.05
Stumbling	0.94±0.31	binary	0.92±0.03	not converged
Shying	1.42±0.87	binary	not converged	0.69±0.18
Teeth-grinding	1.14±0.86	–	not calculated	
Mean rein tension	–	normal	0.61±0.11	0.48±0.14
Maximum rein tension	–	normal	0.71±0.08	0.74±0.08
Rein tension variance	–	normal	0.52±0.14	0.55±0.15
Difference left-right rein	–	normal	0.72±0.27	0.35±0.16
**Rider related**				
Hand aids	6.81±1.20	Poisson	0.13±0.08	0.02±0.03
Leg aids	7.24±1.13	Poisson	0.09±0.06	0.07±0.06
Use of whip	9.95±3.64	Poisson	0.27±0.10	0.11±0.07
Use of voice	6.08±1.82	Poisson	0.31±0.20	0.07±0.05

Mean ± SD of rideability and personality trait scores were calculated along with the Pearson correlation coefficients between these different traits. Subsequently, scores for the personality traits character, temperament and willingness-to-work (excluding the trait constitution due to unavailability of these scores for the mares as well as doubts in how far this trait is indeed a personality rather than health-related trait [4]) as well as rideability were analysed using a linear regression. For this regression a step-wise selection procedure was used to identify, based on the coefficient of determination, of all behavioural and rein tension variables (listed in table 3) those explaining the largest proportion of variance in the respective dependent variable. In addition in a next step, mixed models (parametric data) or generalized linear mixed models (non-parametric data) with a logit link (binary data) or log link (Poisson data) function was used to analyse the effect of categorical factors (horse age [3 or 4 years old], horse gender, location, measurement number [first or second observation], binary behavioural data) as well as the five most influential continuous factors (based on the coefficient of determination from the prior regression analysis) on personality scores. Variables were removed from the model, if they were not significant, and due to their partial confounding, horse gender and location were not considered simultaneously, but one after the other in the analysis. Rein tension and behaviour parameters were analyzed in the same manner. Results from these analyses are presented only, if they were, or tended to be, significant at the P<0.05 or P<0.1 level. To obtain variance components, either horse-rider pairs or only riders were considered in separate runs as a random factor, thus accounting for repeated observations per horse-rider pair or per rider. These variance components were used to calculate repeatabilities on the original scale (normally distributed data [24]) or on the latent scale (Poisson and binary distributed data [25]) for the behavioural and rein tension parameters at the rider as well as the horse-rider pair level. Standard errors of repeatabilities were calculated based on the approximation described e.g. by Roberds and Strom [26]. For the normally distributed traits, significance of random effects was assessed using Chi-Square statistics based on differences in log likelihoods of the mixed models with or without the respective random factor [27]. Due to lacking comparability of log Pseudo-likelihoods of different generalized mixed models, these calculations were not conducted for the non-Gaussian data.

## Results

### Descriptive Statistics and Correlations of Performance Test Scores

The participating horses received in the performance test a mean ± SD rideability score of 7.75±0.65. Scores for the other personality traits were: overall personality = 8.09±0.61, temperament = 8.02±0.41, character = 7.99±0.58, willingness to work = 7.65±0.73, and constitution = 7.88±0.57. Correlation coefficients between rideability and overall personality (r = 0.69; P<0.0001), temperament (r = 0.50; P = 0.0021), character (r = 0.60; P = 0.0001), willingness-to-work (r = 0.56; P = 0.0004) and constitution scores (r = 0.35; P = 0.27) were mostly high and significant. Correlation coefficients among the different personality traits were, again except for the trait constitution, likewise highly significant (P<0.0043) and ranged between 0.44 (temperament – willingness-to-work) and 0.85 (overall personality – character).

### Repeatability of Rein Tension and Behavioural Parameters

Repeatabilities of rein tension parameters were both at the horse*rider level as well as at the rider level within an acceptable range, but repeatabilities varied widely for the behaviour traits (table 3).

### Variance Explained in Rideability and Personality Trait Scores

The regression analysis revealed that a considerable proportion of variance in rideability scores could be explained by rein tension and behavioural parameters. Notably, the three main rein tension parameters (coefficient of determination for maximum tension: 17%, mean rein tension: 16%, and variability of rein tension: 15%) each explained a larger proportion of variance in rideability scores, compared to any behavioural parameter. The maximum value for behavioural parameters was 5% for tail-swishing as well as for rider’s use of hands and horse-induced change in gait, followed by shying (4%). Albeit the overall variance explained was lower, a similar pattern was observed with the willingness to work scores: mean, variance and maximum rein tension explained 9%, 9% and 8% of the variance, respectively, while the three most influential behaviour patterns explained only 4% (involuntary change in gait) or 3% (both snorting and rider’s use of legs) of the variance in willingness to work scores. In contrast, scores for the trait temperament were best explained by the frequency of shying per hour of riding (19%), while maximum rein tension explained a considerably lower proportion of variance (6%), followed by tail-swishing, snorting and the rider’s use of the whip (each 4%). Variance in rein tension explained the largest proportion (12%) of variance in character scores, followed by involuntary change in gait (10%), mean rein tension (9%) and maximum rein tension as well as shying (both 8%).

### Influence of Behaviour and Rein Tension on Rideability and Personality Trait Scores

Rideability scores dropped significantly (all P<0.05) with increasing mean, maximum and variability in rein tension (e.g. −0.37±0.14 scores per additional 1 Newton in mean tension; table 4). In addition, rideability scores tended to drop with increasing frequencies of behaviour patterns such as tail-swishing and shying (table 4).

**Table 4:** Influence of behaviour patterns and rein tension parameters on scores for personality traits.

Personality trait	Physiological/behaviour trait	Influence ± SE	P-values
Rideability (final score)	**Mean rein tension**	**−0.37±0.14**	P = 0.0161
	**Variance in rein tension**	**−0.20±0.08**	P = 0.0205
	**Maximum rein tension**	**−0.14±0.05**	P = 0.0141
	Tail-swishing	−0.15±0.08	P = 0.0871
	Shying	−0.71±0.34	P = 0.0531
	Change in gait	−0.24±0.13	P = 0.0645
	Rider’s use of hands	−0.14±0.08	P = 0.0888
Overall personality	**Mean rein tension**	−**0.51±0.12**	P = 0.0001
	**Variance in rein tension**	−**0.28±0.07**	P = 0.0002
	**Maximum rein tension**	−**0.22±0.04**	P<0.0001
Character	Shying	−0.10±0.05	P = 0.0891
	Change in gait	−0.04±0.02	P = 0.0659
Temperament	**Shying**	−**0.10±0.04**	P = 0.0071
Willingness to work	Mean rein tension	−0.42±0.23	P = 0.0853
	Variance in rein tension	−0.21±0.12	P = 0.0855

The strength of influence indicates the change in personality score per additional occurrence of the behaviour trait during the observation period (bold print = significant relationships at P<0.05; regular print = relationship at P<0.1).

### Other Factors Influencing Rideability Scores, Rein Tension or Behaviour

The random horse*rider effect was significant (P<0.05) for mean and difference in rein tension, indicating that there are considerable differences between horse-rider pairs in the intensity and consistency of tension placed on the rein by the horse and/or the rider. Similarly, the random rider effect was significant for the mean and difference in rein tension, indicating that rein tension is also considerably determined by the rider’s riding style independent of the horse. Mean, maximum and variance of rein tension differed highly significantly between testing stations (P<0.0001) ranging e.g. between a least square mean of 9.1±1.6 N in one station and 18.9±0.9 N and 21.7±1.3 N in the other two stations (table 5). Horses that snorted had lower mean rein tensions than horses that did not snort (P = 0.0074), while rein tension increased as the performance test training progressed: there were lower (P<0.0001) rein tensions observed in the first compared to the second measurement (table 5).

**Table 5:** Factors influencing rein tension parameters.

Parameter	Influencing factor		Influence ± SE	P-values
Mean rein tension	**location**	**A**	**21.7±1.3**	<0.0001
		**B**	**9.1±1.6**	
		**C**	**18.9±0.9**	
	**Measurement number**	**1**	**14.4±0.9**	<0.0001
		**2**	**18.9±0.9**	
	**Snorting**	**0**	**17.3±0.8**	0.0074
		**1**	**12.6±1.6**	
	Change in gait		**0.17±0.1**	0.0306
	Use of whip		−0.08±0.05	0.0882
Variance in rein tension	**location**	**A**	**25.6±3.3**	0.0050
		**B**	**4.45±4.1**	
		**C**	**20.6±3.5**	
Maximum rein tension	**location**	**A**	**90.3±2.4**	<0.0001
		**B**	**61.3±2.9**	
		**C**	**96.5±1.7**	
	**Use of whip**		−**0.14±0.04**	0.0003
	**Use of hand**		**0.22±0.1**	0.0470
Difference left-right rein tension	**Shying**	**0**	−**2.8±0.6**	0.0375
		**1**	**0.89±1.4**	
Relative difference in rein tension	–		–	(all >0.1)

The influence ± SE indicates the least square mean per factor level (categorical variables) or the change in the parameter per increase of the independent variable by one unit (continuous variables). RT = rein tension.

With the exception of bucking, horses’ behaviour was considerably influenced either by the riders’ behaviour and/or rein tension parameters (table 6). In particular, the rider’s use of legs and whip influence a large number of horses’ behaviour patterns. In addition, horse behaviour differed in a few cases by horse gender: stallions were less likely to show tail-swishing (0.95±0.47 times less likely to tail-swish; P = 0.0511) and head-tossing (1.55±0.61 times less likely to toss their head; P = 0.0154) compared to mares. However, the partial confounding of gender with location needs to be kept in mind with these results. There were also considerable relationships between the different behaviour patterns. Horses that shied were also more likely to show horse-induced changes in pace (2.9±0.41 time more likely; P<0.0001) and head-tossing (1.9±0.49 times more likely; P = 0.0004), and they were less likely to attempt to buck (5.0±1.1 times less likely; P<0.0001), compared to horses that did not shy during the observation period. Also, the more often horses tossed their head, the more likely they were to show a horse-induced change in gait (0.05±0.007; P<0.0001) and to tail-swish (0.03±0.006; P<0.0001). Similarly, the more often a horse swished its tail, the more likely it was to head-toss (0.03±0.006; P<0.0001), and the less likely it was to snort (−0.06±0.03; P = 0.0578) and to stumble (−0.07±0.03; P = 0.0246). Horse’s age did not significantly influence any of the behaviour patterns, although the limited number of four-year-olds does not allow for definite conclusions.

**Table 6:** Influence of rider behaviour and rein tension on horses’ behavioural parameters.

Behaviourpattern	Influencing factor	Influence ± SE	P-values
Change in pace	**Use of voice**	**0.04±0.005**	<0.0001
	**Use of leg**	−**0.06±0.02**	0.0105
	**Use of whip**	**0.02±0.003**	<0.0001
	**Maximum rein tension**	−**0.49±0.07**	<0.0001
	**Difference in rein** **tension**	**2.1±0.76**	0.0244
Tail-swishing	**Use of hands**	**0.04±0.01**	0.0005
	Use of whip	0.007±0.004	0.0664
	**Use of leg**	**0.06±0.01**	<0.0001
Head-tossing	**Use of hands**	**0.06±0.01**	<0.0001
	**Use of whip**	**0.02±0.004**	<0.0001
	**Use of voice**	**0.04±0.009**	<0.0001
	**Use of leg**	**0.04±0.01**	0.0007
	Maximum rein tension	−0.15±0.08	0.0756
	Mean rein tension	−0.47±0.26	0.0829
Attempted buck	–	–	(all>0.1)
Stumbling	Use of leg	−0.05±0.03	0.0784
Shying	**Use of whip**	−**0.02±0.008**	0.0365
	**Mean rein tension**	**1.43±0.58**	0.0172
	Maximum rein tension	0.37±0.19	0.0584
	**Variance in rein tension**	**0.87±0.37**	0.0201
Snorting	**Mean rein tension**	**2.08±0.51**	0.0002
	Difference in rein tension	6.75±3.44	0.0856

The influence ± SE indicates the change in probability per increase of the independent variable by one unit.

## Discussion

The traits rideability, temperament, character and willingness-to-work are compound traits with rather vague definitions, each including a large variety of different behaviour patterns. Evaluations of these traits based on scores that classify an animal’s performance in these traits as either “good” or “poor” by different judges that may each have their own set of aspects they focus on during evaluation will thus make it impossible to infer definite, specific behaviour profiles from the scores alone. The present study nevertheless attempted to shed light on some general relationships between rideability and personality trait scores on the one hand and specific behaviour patterns and rein tension parameters on the other hand. Results revealed that the lower and the steadier the rein tension the better judges evaluated horses’ rideability, i.e. the measure of how comfortable it feels to ride a certain horse. Most riding theories request a steady but light contact between the horse’s mouth and the rider’s hand via the reins (e.g. [28]). Also, according to the guidelines [3] as well as a survey, rideability is considered by performance test judges to be partially determined by the intensity and consistency of the rein contact [4]. Thus the relationships between rein tension parameters detected in the present study were expected, and they indicate that performance test judges apparently indeed pay attention to signs indicative of the quality and intensity of rein tension. On the other hand, the rather strong relationships between rein tension and rideability scores are also surprising as riders and judges do not always seem to be particularly good in judging their own rein tension [29] or in agreeing on the lightness of riders’ aids [30], respectively. In addition, the insufficiencies of the current rideability and personality evaluation methods have been highlighted repeatedly [4], [7], [12], [31], and therefore, the identified relationships may not be particularly meaningful. For example, these relationships may not exist when using a different set of judges. However, rideability scores from the performance test are an accepted measurement in practice and are at present the only available data on this parameter. In future studies it would be interesting to consider the individual scores assigned by the coach, the judge from the ground and the test rider separately to investigate potential differences in their evaluation strategies.

The present study is the first to report repeatabilities of rein tension and behavioural parameters assessed in the ridden horse both at the rider as well as the horse-rider level. Repeatabilities for rein tension parameters and some, but not all behavioural parameters were remarkably high, and within similar ranges for horse-rider pairs as well as riders. However, the sample size of the present study was small, and it would be important to confirm the results in a larger sample of horses. Repeatabilities for the rein tension parameters compare well to values for horses’ reactivity in standardized temperament tests [12] as well as to performance parameters [32], [33]. Therefore, these moderately to highly repeatable traits potentially qualify for future, large-scale investigations such as are required for the estimation of genetic parameters. Furthermore, considering both the comparably high repeatabilities of rein tensions parameters and the relation between rein tension parameters and rideability scores, results from the present study indicate that the evaluation of at least some aspects of rideability could be made more objective, if direct measures of rein tension were taken instead of subjective scores. However, rein tension parameters alone will not be sufficient as it is not possible based on the plain values to distinguish a desired, very light rein contact from horses’ avoidance of rein contact (i.e. the horse going “behind the bit”). Additional recording of the horses’ behaviour and head posture will be indispensable for proper interpretation of the rein tension measurements.

Furthermore, at the present stage, the technical equipment may not yet be robust enough, and in general dependence on the technical equipment may be prohibitive for introduction in performance tests. For example in an earlier version of this experiment, no rein tension measurements could be obtained as the rein gauges broke within minutes of testing the first horse due to overload of the sensors which had a maximum capacity of 50 N. Although more powerful sensors (maximum capacity of 100 N) were used for the present experiment, and no further problems disturbed the measurements of the present study, this incidence of equipment failure serves as an example of the susceptibility of technical, equipment-based measurements to data loss. Other potential sources of data loss include failure of power supply, memory card, or hardware, and these potential sources of data loss must be minimized before performance test evaluations can be replaced by technological devices. Alternative solutions such as the subjective assessment of rein tension by specifically trained judges, or the use of indicator traits such as rein length [34] may not yield satisfactory results either. Continued reliance on judges’ evaluations would not overcome the problems inherent to subjective evaluations [4], and rein length is a parameter influenced directly and almost unilaterally by the rider and may thus not be suitable to evaluate horses’ innate characteristics.

Lower proportions of variance in rideability scores explained by behaviour patterns in the present study compared to the previous study [9] may be a result of different judges evaluating the traits at the different locations. It is likely that, due to the rather subjective evaluation criteria [4] along with the large amount of individual factors that comprise the present, complex traits, each judge has her/his own aspects she or he focuses on during evaluation of personality traits as well as rideability. Combining the results from these different judges will thus lower the impact of relationships that exist within parameters evaluated by one judge. In addition, these values have to be seen in light of the suboptimal statistical properties of both the behavioural parameters as well as the rideability scores [13]. Direct comparisons between e.g. behaviour and rein tension parameters with different distributions suboptimal for regression analysis should be considered with care, and if there is overall little variance present, it may be easier to explain a significant proportion of this limited variance. Means and standard deviations for rideability (7.8±0.7) and personality scores (e.g. temperament: 8.0±0.4) were similar to scores obtained in the recent past by larger groups of performance tested horses in Poland [7] between 2004 and 2007 or in Germany (7.8±0.9 and 8.3±0.8 for temperament and rideability, respectively between 2007 and 2010 [13]). The high, phenotypic correlations found in the present study between the different personality traits are also typical of performance test scores [12]. Therefore, the horses used in the present study appear to be a representative sample of the general participants of performance tests. These high correlations between the different personality trait scores once again highlight the insufficiencies of the present personality trait evaluation system, and also explain why a larger number of behaviour and rein tension traits in present study simultaneously explain significant amounts of variation in different personality traits (e.g. rein tension parameters significantly influence rideability scores as well as overall personality scores and character scores [see table 5]).

Mean rein tension is with ca. 9–20 N comparable to results from earlier studies investigating similar riding situations [35]–[39]. However, mean rein tension in all these studies was remarkably high and considerably higher than the tension young horses would accept voluntarily (ca. 6–10 N; [40]). Thus, not surprisingly, in the present as well as in the above-mentioned study [40] higher rein tensions were associated with higher levels of potential conflict behaviour such as horse-induced change in gait [36], and with lower levels of potential comfort behaviour such as snorting. More frequent shying was also associated with higher mean and maximum rein tension, although as with any of these statistical relationships cause and effect are not clear. Possibly, riders tried to restrain horses more strongly after they shied, but possibly, horses ridden with stronger rein contact were more fearful and thus showed shying more frequently. Such an enhancing effect of more coercive riding techniques was shown earlier [41] and may be the result of additive effects of anxiety on fear reactions [42]. In contrast, the frequency of head-tossing tended to be reduced with increasing mean and maximum rein tensions, potentially because the high rein pressure physically prevented the horses from exhibiting any potential discomfort or avoidance behaviour via head movements for fear of yet increasing the pressure in the mouth. Ineffectiveness of or inability to express avoidance behaviour has the potential to provoke a state of learnt helplessness [43]. In this context heightened rein tensions potentially have to be considered as a severe threat to equine welfare. A stronger focus on the evaluation of rein tension during horse shows appears to be a logical step to advocate the ridden horses’ welfare.

Differences between rein tension in left and right reins likely are the combined result of horses’ and riders’ laterality [35]. Minimizing lateralisation is an important aspect in training of young horses, which is why associations with personality evaluations were expected, but not confirmed by the present study. The association between absolute difference in rein tension and shying potentially relates to horses’ emotional laterality, i.e. their preferences for a certain eye when observing frightening objects [44].

Striking differences in mean, maximum and variance in rein tension between test stations indicate that there may be differences in “riding culture” maintained e.g. by the head coach, that lead to marked differences in the amount of force applied on the reins. Differences in individual riders’ riding styles, e.g. regarding the differences in their use of visible hand aids and the use of whips further support this view. Although the confounding of gender with locations makes conclusions regarding these effects difficult, generally the location appears to play the more important role. With more parameters, one of the two mare stations rather than the stallion station differed significantly from the other two locations. Such differences in local “riding culture” were also observed for different riding schools [45], but are nevertheless surprising in the present study: performance tests should provide as much as possible standardized training conditions in order to allow for an unbiased assessment of the animals’ genetic merit. Thus, those factors clearly need to be standardized or controlled for, when using rein tension measurements in evaluation of horses’ rideability. Furthermore, the significant influence of the rider as well as the horse-rider pair underlined that mean rein tension is determined by both the horse and the rider. Likewise, the considerable influence of riders’ behaviour on horses’ behaviour demonstrates that riders indirectly have an impact on horses’ evaluation in the performance test. Repeated evaluation of rideability under different riders would have been interesting to further shed light on the influence the rider has on rideability evaluations. However, due to the study’s set up within the official performance test training, it was not possible to change training conditions by using different riders per horse. The continued use of independent test riders, whose individual level of rein tension and intensity of aids will be known from repeated observations and can thus be corrected for as is the case during genetic evaluations based on sport horse data [46], could be a potential solution. Nevertheless, interaction effects between specific horses and riders also exist, such that a given horse-rider combination matches particularly well or poorly and will thus yield particularly low or high values in rein tension. Unfortunately, such interaction effects that could indicate which type of horse is particularly suitable for a certain type of rider/riding style could only be tested reliably in a very large number of horses ridden by several different riders. The routine evaluation by two different test riders during the performance test would be an important step into this direction.

Overall, the results of the present study confirm the insufficiencies of the present personality and rideability evaluation system in horse breeding. The vague definitions of very complex traits do not allow for objective and transparent evaluations. This is also reflected by a lack of associations between behaviour patterns that should, according to the guidelines, be related to rideability. Defining a precise list of behaviour patterns whose frequencies can be counted or whose intensities can be measured is an important step towards an improvement of the situation. However, in order to assign meaning to such measurements the specific context of behaviour patterns may have to be considered, which is a limitation of the present study. Although performance tests are intended to solely evaluate the animals’ abilities, the present study supports common knowledge that riders considerably influence the horses’ traits. These findings highlight the importance that the rider’s actions need to be recorded, too. These record would allow for both evaluation of the horse’s reaction to the rider’s aids as well as statistical corrections for the influence of the rider’s riding style when evaluating horse’s rideability. Ultimately such a revised evaluation system would allow breeders to make more informed decisions when selecting stallions for their mares based on rideability aspects.
